# Changes in Neuropathic Pain Profiles by Dysesthesia-Matched Transcutaneous Electrical Nerve Stimulation: A Case Report

**DOI:** 10.7759/cureus.94322

**Published:** 2025-10-11

**Authors:** Takashi Hoei, Yuki Nishi, Seiji Etoh, Kentaro Kawamura, Megumi Shimodozono

**Affiliations:** 1 Division of Rehabilitation, Kagoshima University Hospital, Kagoshima City, JPN; 2 Institute of Biomedical Sciences (Health Sciences), Nagasaki University, Nagasaki City, JPN; 3 Department of Rehabilitation and Physical Medicine, Kagoshima University Graduate School of Medical and Dental Sciences, Kagoshima City, JPN

**Keywords:** cervical spinal stenosis, neuropathic pain, rehabilitation, sensorimotor function, transcutaneous electrical nerve stimulation

## Abstract

Transcutaneous electrical nerve stimulation (TENS) is widely used to treat neuropathic pain because of its safety, but its therapeutic efficacy is limited. Dysesthesia-matched TENS (DM-TENS) is an innovative intervention that synchronizes TENS parameters with an individual patient's perception of dysesthesia. This case report describes a patient with cervical spinal stenosis whose throbbing pain evolved into dysesthesia. DM-TENS was synchronized with each neuropathic pain profile. Initially, the patient’s throbbing pain was rated 8 on a numerical rating scale (NRS), and the pinch force adjustment test (pinch test) showed a 2.58% mean error. DM-TENS synchronized to throbbing pain immediately reduced the NRS to 2 and the pinch test score to 2.06%. On postoperative day (POD) 13, the throbbing pain disappeared and evolved into dysesthesia (NRS 5, pinch test 2.41%). When the DM-TENS parameters were adjusted to match dysesthesia, immediate improvement was also achieved (NRS 0, pinch test 1.69%). By POD 20, the NRS for dysesthesia decreased to 3, and the patient reported high satisfaction. To our knowledge, this is the first case to demonstrate the effectiveness of DM-TENS synchronized with pain characteristics other than dysesthesia, highlighting the potential to broaden its clinical applications.

## Introduction

Neuropathic pain is caused by lesions or diseases of the somatosensory system [1]. It is known to be common in patients with spinal cord-associated diseases and has been shown to reduce quality of life [2]. Neuropathic pain associated with spinal cord-related diseases presents with a wide range of sensory characteristics. In particular, throbbing pain is considered to be a painful condition that causes widespread disruption to daily life and an effective treatment for this condition is required [1-3].

Transcutaneous electrical nerve stimulation (TENS), a non-pharmacological therapy, is widely used due to its safety profile; however, its therapeutic efficacy remains limited [4]. Recently, Nishi et al. developed dysesthesia-matched TENS (DM-TENS) as an innovative intervention that synchronizes TENS parameters with an individual's specific dysesthesia [5]. The specific method of synchronization involves first adjusting the stimulation intensity to correspond to the perceived intensity of dysesthesia and then matching the TENS frequency to the subtle sensory sensations associated with dysesthesia, such as tingling or pins and needles. Although DM-TENS requires individualized parameter adjustments for each patient’s unique sensory profile, this personalized approach has been shown to produce greater relief of dysesthesia and even enhance tactile perception compared with conventional TENS [5]. DM-TENS is synchronized to the dysesthesia sensation, but it has also been shown to be effective for other pain characteristics (e.g., tingling or pins and needles, numbness, allodynia, and electrical shock pain) [5,6]. However, some patients with neuropathic pain do not experience dysesthesia. To date, there have been no reports of DM-TENS parameters being set to characteristics other than dysesthesia, such as throbbing pain, and its use is expected to expand.

This case report presents a patient with cervical spinal stenosis. Initially, the patient experienced throbbing pain in the left hand without dysesthesia, which later progressed to dysesthesia. DM-TENS was synchronized with both neuropathic pain profiles, throbbing pain and dysesthesia, and comprehensive assessments of neuropathic pain and sensorimotor function were conducted.

## Case presentation

Patient information

**Figure 1:**
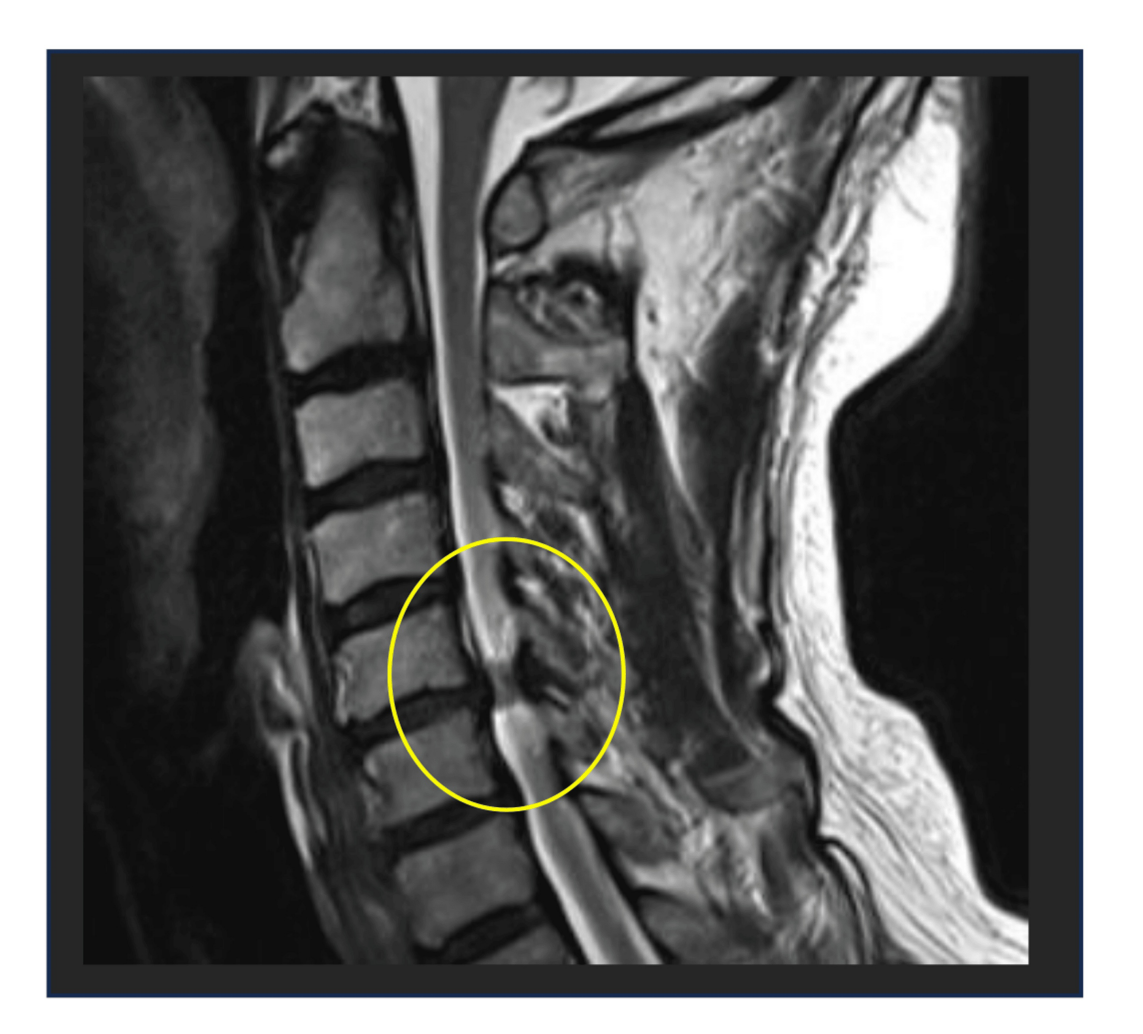
Preoperative T2-weighted MRI of the cervical spine Sagittal T2-weighted MRI showing spinal cord compression at C5/6. MRI, magnetic resonance imaging.

A 66-year-old right-handed man first noticed difficulty moving his left leg one year ago and visited his previous doctor eight months later. He was diagnosed with spinal canal stenosis and myelopathy and was receiving conservative treatment. Six months ago, he began to experience numbness in his left hand, which progressed to dull pain three months ago. Due to worsening symptoms, he was referred to the orthopedic department of our hospital for surgery one month ago and was hospitalized. An MRI revealed spinal canal stenosis at the C5/6 level (Figure 1), and the patient underwent C3 laminectomy and C4-6 laminoplasty. His medical history included diabetes mellitus and dyslipidemia. The rehabilitation department became involved after the surgery, and occupational therapy was initiated on the first postoperative day (POD). The occupational therapy interventions comprised assisted sitting and bed mobility following surgery, gait training, and range-of-motion exercises aimed at promoting early postoperative recovery.

In the international standards for the neurological classification of spinal cord injury [7], motor and sensory function following spinal cord injury were assessed only in the upper limbs. The motor score (maximum score of 25) was 25 points on the right and 21 points on the left (C5: 4, C6: 5, C7: 4, C8: 4, T1: 4). The light touch score (maximum score of 10) was 10 on the right and 8 on the left (C5: 1, C6: 1, C7: 2, C8: 2, T1: 2), and the pin prick score (maximum score of 10) was also 10 on the right and 8 on the left (C5: 2, C6: 0, C7: 2, C8: 2, T1: 2). Occupational therapy included upper and lower limb exercises, and gait training. The patient was able to walk independently on POD 9. The median and ulnar nerves were electrically stimulated at the wrist, and somatosensory evoked potentials were recorded from the contralateral sensory cortex on the scalp. The N20 latencies were prolonged beyond the normal range bilaterally, with no left-right difference, while the amplitudes remained within normal limits (Figure 6, Appendix). However, severe pain in the left hand persisted (numerical rating scale (NRS): 8). The patient described the pain in his left hand as a throbbing pain, like a razor cut, and held his left hand in front of his chest with his right hand. However, this throbbing pain was not the typical slow, pulsating type; rather, it was fast-paced and continuous, with no pauses between the waves of pain. Therefore, we defined this patient’s pain as “rapid, persistent, throbbing (RPT) pain.” He also reported a pressure-like pain sensation at the tip of his index finger, as if it were being pinched. Following the rehabilitation physician’s instructions, we initiated DM-TENS to relieve the pain. This case report was approved by the clinical research Ethics Committee of Kagoshima University Hospital (No. 240306). The patient provided written consent for publication of the case report.

Intervention

Dysesthesia-Matched Transcutaneous Electrical Nerve Stimulation (DM-TENS)

**Figure 2:**
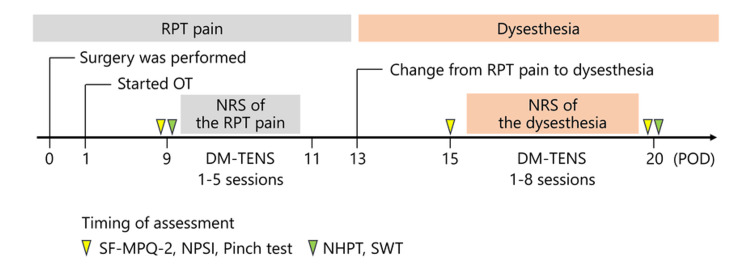
Timeline illustrating the overall clinical course, from surgery through symptom changes to DM-TENS intervention The characteristics of neuropathic pain are shown in the upper panel, and events and assessment timing are shown in the lower panel. RPT pain, rapid, persistent, throbbing pain; OT, occupational therapy; POD, postoperative day; NRS, numerical rating scale; SF-MPQ-2, short-form McGill pain questionnaire; NPSI, neuropathic pain symptom inventory; Pinch test, pinch force adjustment test; NHPT, nine-hole peg test; SWT, Semmes-Weinstein monofilament test; DM-TENS, dysesthesia-matched transcutaneous electrical nerve stimulation.

**Figure 3:**
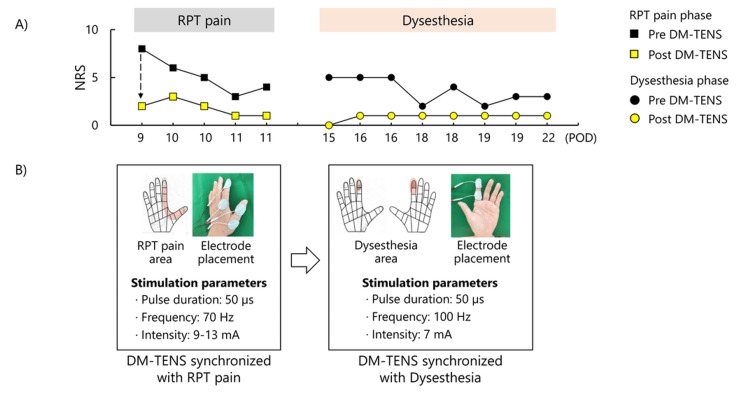
Time course of NRS changes and DM-TENS stimulation parameters A) Time course of NRS scores for RPT pain and dysesthesia across the intervention period. In both phases, black symbols represent pre-DM-TENS scores, and yellow symbols represent post-DM-TENS scores. The RPT pain phase included five sessions over three days, and the dysesthesia phase included eight sessions over eight days. B) Pain drawings illustrate the areas of symptoms, and photographs show electrode placements for DM-TENS. Stimulation parameters were as follows: RPT pain phase (pulse duration 50 µs, frequency 70 Hz, intensity 9-13 mA) and dysesthesia phase (pulse duration 50 µs, frequency 100 Hz, intensity 7 mA). Each panel summarizes the symptom progression in each RPT pain and dysesthesia phase, along with the corresponding stimulation settings. RPT, rapid, persistent, throbbing pain; POD, postoperative day; DM-TENS, dysesthesia-matched transcutaneous electrical nerve stimulation; NRS, numerical rating scale.

Figure 2 shows a timeline summarizing the patient’s clinical course from surgery to DM-TENS intervention. The patient's neuropathic pain initially presented with RPT pain characteristics, which developed into dysesthesia after POD 13 (Figure 3A).

Electrical stimulation for DM-TENS was performed using a low-frequency therapy device (Espurge, Ito Physiotherapy, and Rehabilitation Co., Japan). Small circular electrodes (32 mm in diameter, gel pad, Ito Physiotherapy, and Rehabilitation Co., Japan) were applied directly to the painful area over the left thumb and index finger, as identified by the pain drawing (Figure 3B) [8]. DM-TENS was delivered using a continuous pulse pattern with a 50-µs pulse duration and symmetrical waveform. The stimulation frequency was adjusted to match the beats of the patient’s spontaneous RPT pain, and the intensity was set to correspond to the perceived pain strength [5,6]. DM-TENS was performed for 20-30 minutes per session, with five sessions conducted over three days during the pain phase (twice daily for two days and once on one day). Stimulation parameters included a frequency of 70 Hz and an intensity of 9-13 mA.

Assessment

The patient experienced RPT pain in the index finger and thumb of the left hand and complained of difficulty using the hand.

**Figure 4:**
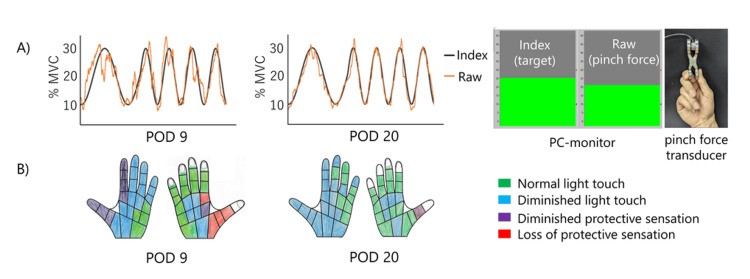
Pinch test and SWT A) Representative waveforms from the pinch test at POD 9 and POD 20, showing improved force adjustment. The black line represents the index, and the orange line represents the actual pinch force (raw). The patient was instructed to adjust the pinch force so that the ‘raw’ marker on the right followed the up-and-down movement of the index displayed on the PC monitor. The pinch test was performed using a device developed by Applied Office (Tokyo, Japan). B) Sensory evaluation using the SWT. Colored hand maps illustrate the degree of tactile perception: green = normal, blue = diminished light touch, purple = diminished protective sensation, red = loss of protective sensation. %MVC, maximum voluntary contraction; Pinch test, pinch force adjustment test; SWT, Semmes-Weinstein monofilament test; POD, postoperative day.

Neuropathic pain was assessed using the short-form McGill pain questionnaire version-2 (SF-MPQ-2) [9], which evaluates the characteristics and intensity of pain, and the neuropathic pain symptom inventory (NPSI) [10], which evaluates the severity of neuropathic pain. The SF-MPQ-2 consists of 22 items, including 18 sensory items and four affective items, and is designed to comprehensively assess both the quality and intensity of pain [11]. It provides valuable insight into the sensory profile of neuropathic pain across different phases. The sensory items include: 1. throbbing pain, 2. shooting pain, 3. stabbing pain, 4. sharp pain, 5. cramping pain, 6. gnawing pain, 7. hot-burning pain, 8. aching pain, 9. heavy pain, 10. tender, 11. splitting pain, 12. tiring-exhausting, 13. sickening, 14. fearful, 15. punishing-cruel, 16. electric-shock pain, 17. cold-freezing pain, 18. piercing, 19. pain caused by light touch, 20. itching, 21. tingling or ‘pins and needles’, and 22. numbness. Pain intensity was also evaluated using the NRS. The SF-MPQ-2 and NPSI were evaluated on POD 9, POD 15, and POD 20. The NRS was evaluated before and after DM-TENS intervention. To evaluate precision pinch force control, the pinch force adjustment test (pinch test) was performed on POD 9, POD 15, and POD 20 [12]. The pinch test assesses tracking error, which reflects the ability to control force during fine motor tasks (Figure 4A). In addition, sensory function was evaluated on POD 9 and POD 20, using the Semmes-Weinstein monofilament test (SWT) [13], and object manipulation ability was assessed using the nine-hole peg test (NHPT) [14].

Results

Table 1 summarizes the clinical assessment results across the intervention period, including pain score, sensory and motor tests, and patient satisfaction. The patient reported that DM-TENS during the RPT pain phase "reduced the pain and made me feel better." Satisfaction with the treatment was high, with a score of 8 on a 10-point NRS (0 = not satisfied, 10 = extremely satisfied). Figure 3A shows the intensity of neuropathic pain before and after treatment.

**Table 1:** Clinical assessment results across the intervention period Results of the SWT are illustrated in Figure 4. POD, postoperative day; DM-TENS, dysesthesia-matched transcutaneous electrical nerve stimulation; SF-MPQ-2, short-form McGill pain questionnaire; NPSI, neuropathic pain symptom inventory; Pinch test, pinch force adjustment test; NHPT, nine-hole peg test; NRS, numerical rating scale; RPT pain, rapid, persistent, throbbing pain; —, not assessed; SWT, Semmes-Weinstein monofilament test.

Outcome measure	POD 9		POD 15		POD 20	
	pre DM-TENS	post DM-TENS	pre DM-TENS	post DM-TENS	pre DM-TENS	post DM-TENS
SF-MPQ-2 (score)	51	3	33	—	11	—
NPSI (score)	23	—	25	—	11	—
Pinch test (%)	2.58	2.06	2.41	1.69	1.84	1.56
NHPT (time)	32.6	—	—	—	25.7	—
Satisfaction (NRS)	—	—	8	—	9	—
RPT pain (NRS)	8	2	—	—	—	—
Dysesthesia (NRS)	—	—	5	0	3	1

**Figure 5:**
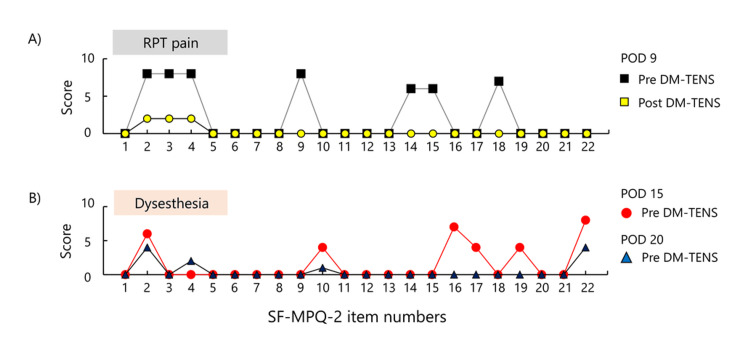
Changes in neuropathic pain profiles assessed by SF-MPQ-2 Neuropathic pain was assessed using the SF-MPQ-2. The X and Y axes represent the item number and the score of the SF-MPQ-2, respectively. A) Scores before and after DM-TENS on POD 9 (initiation of DM-TENS) during the RPT pain phase. B) Scores before DM-TENS on POD 15 and POD 20 during the dysesthesia phase. Changes in neuropathic pain profiles during the RPT pain and dysesthesia phases are shown. Panel A illustrates the immediate effect of DM-TENS on POD 9, while Panel B presents the changes observed on PODs 15 and 20. SF-MPQ-2, short-form McGill pain questionnaire; RPT pain, rapid, persistent, throbbing pain; POD, postoperative day; DM-TENS, dysesthesia-matched transcutaneous electrical nerve stimulation; NRS, numerical rating scale.

**Figure 6:**
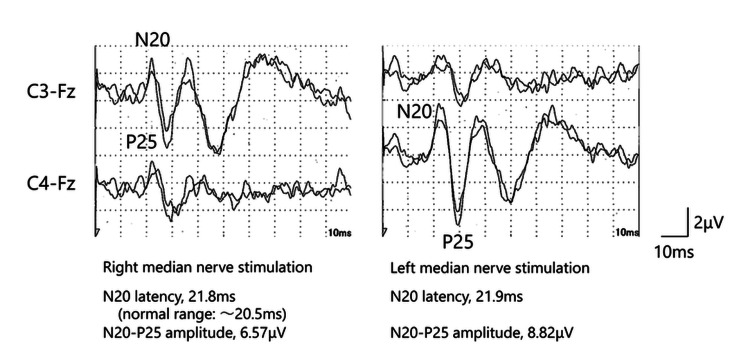
Somatosensory evoked potential Electrical stimuli were applied to the right median nerve for 0.2 ms at the wrist. Stimulus intensity was just above motor threshold (6.6 mA on the right and 6.0 mA on the left). Stimuli were presented at 3 Hz. All electrophysiological recordings were acquired using the Neuropack MEB-2200 (Nihon Kohden, Tokyo, Japan). EEG was performed using Ag/AgCl electrodes placed over two scalp sites, C3′ (2 cm posterior to C3) and C4′ (2 cm posterior to C4), according to the International 10–20 system. A reference electrode was placed at Fz. EEG signals were recorded with a 20–3000 Hz bandpass filter. The SEP analysis period ranged from 0 ms to 100 ms after stimulus onset. We analyzed the latency and peak-to-peak amplitudes of the two cortical SEP components (N20, P25). An average of 500 recordings was obtained. SEP, Somatosensory evoked potential; EEG, Electroencephalography

On POD 13, the patient noticed that the pain had disappeared and was replaced by a dysesthesia sensation. Accordingly, the clinical course was divided into two phases: an RPT pain phase and a dysesthesia phase. Figure 5 presents the change in the sensory characteristics of pain between the RPT pain phase and the dysesthesia phase, as measured by all items of the SF-MPQ-2. A pain drawing revealed that the area of dysesthesia was localized to the tip of the index finger (Figure 3B). Accordingly, the electrode placement for DM-TENS was adjusted to the index finger (Figure 3B), and the stimulation parameters were also adjusted to match the beats with the dysesthesia. DM-TENS was performed for 20-30 minutes per session, with eight sessions conducted over eight days during the dysesthesia phase. The stimulation parameters during the dysesthesia phase were different from those used in the RPT pain phase (70 Hz, 9-13 mA): set at a frequency of 100 Hz and an intensity of 7 mA (Figure 3B). The dysesthesia decreased with the use of DM-TENS. On POD 20, after completing DM-TENS sessions, the patient reported, "It's comfortable because there's no pain. I can live with this level of dysesthesia." On POD 20, satisfaction remained high, with a score of 9 on a 10-point NRS. The NPSI scores were as follows: POD 9: 23 points, POD 15: 25 points, and POD 20: 11 points. Pinch tests were performed twice before and after each treatment phase. The pinch test, calculated as the average difference between the target and actual force, was as follows: POD 9: pre DM-TENS = 2.58%, post DM-TENS = 2.06%, POD 15: pre DM-TENS = 2.41%, post DM-TENS = 1.69%, POD 20: pre DM-TENS = 1.84%, and post DM-TENS = 1.56%. Figure 4B presents the SWT results, illustrating a change in tactile sensation between POD 9 and POD 20. On POD 9, the thumb and index finger exhibited multiple areas classified as "loss of protective sensation" or "diminished protective sensation". By POD 20, the "loss of protective sensation" had resolved, and most areas demonstrated either "normal light touch" or "diminished light touch," with only a few areas remaining as "diminished protective sensation". NHPT performance was as follows: POD 9: 32.6 seconds, POD 20: 25.7 seconds.

No adverse events occurred during the intervention period.

## Discussion

In this case, DM-TENS was administered to a patient with RPT pain without accompanying dysesthesia. DM-TENS provided immediate pain relief, and by POD 13, the nature of the pain had shifted from RPT to dysesthesia. When the DM-TENS parameters were adjusted to match the dysesthesia, immediate relief was achieved. Continued DM-TENS sessions resulted in further improvement in neuropathic pain and sensorimotor function by POD 20, and the patient reported high satisfaction with the intervention. To our knowledge, this is the first case report to suggest that neuropathic pain can be alleviated by adjusting DM-TENS parameters to match pain characteristics other than dysesthesia. These findings suggest the potential to broaden the indications for DM-TENS.

Immediately after the first DM-TENS intervention (POD 9), RPT pain in the patient showed immediate improvement and decreased as the intervention progressed. Moreover, several items on the SF-MPQ-2, representing intermittent pain (items 2, 3, 4, 18), continuous pain (item 9), and affective pain (items 14, 15), were significantly reduced or eliminated immediately following the intervention. However, the score for item 1 “throbbing pain” was 0 even at baseline. Although the patient described the pain as “throbbing”, he clarified that it was not pulsatile or rhythmic but rather a sustained, fast-paced sensation. This discrepancy likely accounts for the low score on item 1, as it did not align with the definition of “throbbing pain” in the Japanese version of the SF-MPQ-2 [9] used in this case report. Mirza et al. previously reported that throbbing pain often lacks synchronization with the arterial pulse [3], which may further explain the mismatch between the patient’s description and the standardized item score. Not only was the RPT pain progressively alleviated, but it also completely disappeared by POD 13, at which point it was replaced by a sensation of dysesthesia. In addition, the area became more localized, shifting from the thumb and index finger to just the tip of the index finger. This change in symptomatology was also reflected in corresponding alterations in the SF-MPQ-2 items score. During the RPT phase, the SF-MPQ-2 items reflected persistent pain, intermittent pain, and affective pain. However, in the dysesthesia phase, the affective pain subsided, new sensory characteristics of neuropathic pain emerged, and the associated affective distress was alleviated, with the patient reporting a sense of comfort.

The exact mechanism underlying this change in pain characteristics remains unclear. Moriwaki et al. described a case in which a patient with chronic pain experienced changes in sensation [15]. Initially, the patient exhibited both allodynia and dysesthesia extending from the thumb to the middle finger. As symptoms improved with treatment, the allodynia resolved, and the previously painful area was subsequently experienced as dysesthesia. Furthermore, it has been suggested that a reduction in the area of tactile paresthesia may occur concurrently with pain relief. Such changes might originate from altered processing within the central nervous system, whereby changes in the overall balance of pain input dynamically modulate the receptive fields of somatosensory neurons, resulting in fluctuating paresthesia symptoms.

Regarding the recovery of sensorimotor function of the hand, the improvement in tactile perception is consistent with previous findings [5,6]. However, the pinch test and NHPT, both of which assess hand dexterity, represent outcome measures that have not been previously examined in the context of DM-TENS. In particular, the pinch test, as a quantitative measure, enabled assessment of the patient's force control [12]. During the RPT pain phase (POD 9), the patient's pinch test score before the intervention was outside the normal range (mean: 1.86%, SD ± 0.45, based on 37 healthy controls). The patient reported that the pain intensified with increasing force. However, immediately after DM-TENS, the score normalized, and the patient reported both pain relief and that the task had become easier to perform. During the dysesthesia phase (POD 15), the patient's pinch test score before the intervention was slightly outside the normal range. Although the patient did not report any pain, he noted that dysesthesia made it difficult to perceive force. However, immediately after DM-TENS, the score normalized, and the patient reported that the reduction in dysesthesia made the task easier to perform. At the final evaluation (POD 20), the score remained within the normal range. TENS has been shown to induce transient sensory abnormalities and elevated mechanical perception thresholds through artificial afferent input, potentially introducing noise into sensory information [16] and short-term declines in sensorimotor integration [17]. In contrast, DM-TENS synchronized stimulation parameters with abnormal sensations such as RPT pain and dysesthesia, effectively canceling out both the sensory abnormalities and the sensation of electrical stimulation. Patients reported a "canceled each other out" phenomenon, which supports previous findings [5]. Nishi et al. suggested that this phenomenon may result from the selective suppression of spinal cord excitability via the so-called “busy line” effect [5]. In other words, we hypothesize that RPT pain and dysesthesia were attenuated, leading to improved tactile perception and enhanced performance on the pinch test, which requires sensorimotor integration. Recently, Shoka et al. proposed that DM-TENS may not only relieve dysesthesia but also improve postural control in patients with plantar dysesthesia due to lumbar spinal stenosis [18]. These findings highlight the potential of DM-TENS as a novel approach to modulate sensorimotor integration, warranting further investigation. The NHPT is commonly used to assess hand dexterity, although it has been reported that performance may be influenced by factors such as pain, dysesthesia, and force control [19]. In this case, the NHPT completion time improved at the final evaluation (POD 20) compared to the initial evaluation (POD 9), suggesting enhanced hand dexterity. These improvements in quantitative outcomes, such as the pinch test and NHPT, likely reflect enhanced fine motor coordination and tactile feedback, suggesting that the improved sensorimotor integration achieved by DM-TENS had a positive impact on the recovery of hand use in daily life.

This case report is limited to a single patient, making it difficult to generalize the findings to the broader pathological mechanisms of neuropathic pain. Furthermore, because DM-TENS was administered as part of acute postoperative rehabilitation, occupational therapy was conducted in parallel to promote functional recovery. Therefore, it cannot be denied that occupational therapy may have influenced the recovery of neuropathic pain. However, although the immediate and reproducible changes observed after DM-TENS suggest a specific effect of DM-TENS, the potential effects of practice and expectation cannot be completely ruled out. Because this case involved the acute postoperative phase, detailed assessment of activities of daily living in the patient’s home environment was not feasible. Additionally, as the patient was admitted from a distant location, long-term follow-up to evaluate sustained effects could not be performed. As this is a single case report, the study design inherently limits generalizability. Future studies should include a larger number of cases and incorporate objective functional assessments to validate these results.

## Conclusions

This case demonstrates that DM-TENS, when tailored to distinct neuropathic pain profiles (RPT pain and dysesthesia), can achieve comprehensive improvement in both neuropathic pain and sensorimotor outcomes following cervical spinal stenosis surgery. These results highlight the potential of DM-TENS to extend beyond dysesthesia-specific applications and broaden its clinical utility. However, DM-TENS synchronized with the RPT pain profile has never been reported before, so the results of this case report may need careful interpretation. In addition to accumulating a larger number of cases, further studies using case series and single-case designs are warranted.
